# Identification of a Human Anti-Alpha-Toxin Monoclonal Antibody Against *Staphylococcus aureus* Infection

**DOI:** 10.3389/fmicb.2021.692279

**Published:** 2021-07-15

**Authors:** Fangjie Liu, Zhangchun Guan, Yu Liu, Jingjing Li, Chenghua Liu, Yaping Gao, Yuanfang Ma, Jiannan Feng, Beifen Shen, Guang Yang

**Affiliations:** ^1^Beijing Institute of Pharmacology and Toxicology, Beijing, China; ^2^State Key Laboratory of Toxicology and Medical Countermeasures, Beijing, China; ^3^Laboratory of Cellular and Molecular Immunology, Institute of Immunology, Henan University, Kaifeng, China

**Keywords:** alpha-toxin, human monoclonal antibody, *Staphylococcus aureus*, infection disease, epitope mapping

## Abstract

*Staphylococcus aureus* is a major pathogenic bacterium that causes a variety of clinical infections. The emergence of multi-drug resistant mechanisms requires novel strategies to mitigate *S. aureus* infection. Alpha-hemolysin (Hla) is a key virulence factor that is believed to play a significant role in the pathogenesis of *S. aureus* infections. In this study, we screened a naïve human Fab library for identification of monoclonal antibodies targeting Hla by phage display technology. We found that the monoclonal antibody YG1 blocked the Hla-mediated lysis of rabbit red blood cells and inhibited Hla binding to A549 cells in a concentration-dependent manner. YG1 also provided protection against acute peritoneal infection, bacteremia, and pneumonia in murine models. We further characterized its epitope using different Hla variants and found that the amino acids N209 and F210 of Hla were functionally and structurally important for YG1 binding. Overall, these results indicated that targeting Hla with YG1 could serve as a promising protective strategy against *S. aureus* infection.

## Introduction

*Staphylococcus aureus* is an important pathogen that causes a diverse array of illnesses, from minor skin infections to life-threatening diseases, such as sepsis and pneumonia (Beceiro et al., 2013; Thammavongsa et al., 2013; Monaco et al., 2017; Lakhundi and Zhang, 2018). Antibiotics are the standard treatment for *S. aureus* infections; however, due to the rapid development of antibiotic-resistant *S. aureus*, treatment is complicated (Gordon and Lowy, 2008; Harris et al., 2010; Espadinha et al., 2013). *S. aureus* pneumonia is associated with mortality rates as high as 60% (David and Daum, 2010), and with the emergence of resistance to glycopeptides, the mortality rate in pneumonia patients treated with vancomycin remains high. In order to avoid the spread of antibiotics resistance, researchers have focused on developing novel strategies to mitigate *S. aureus* infection (Tkaczyk et al., 2012; Oganesyan et al., 2014; Lehar et al., 2015; Liu et al., 2015).

Extracellular toxins play a significant role in the pathogenesis of *S. aureus* infection. Inhibition of toxins is thought to provide less selective pressure for the development of resistance compared to killing bacteria or preventing their growth. Alpha-hemolysin (Hla), which is expressed by most *S. aureus* clinical isolates, is a major extracellular toxin that contributes to pneumonia, dermonecrosis, endocarditis, and sepsis (Bayer et al., 1997; Kennedy et al., 2010; Kebaier et al., 2012; Powers et al., 2015). Hla forms a heptameric pore to penetrate the cell membrane, which induces cell injury and death. A disintegrin and metalloprotease 10 (ADAM10) has been identified as the cellular receptor, which is critical for cell lysis mediated by Hla (Wilke and Bubeck Wardenburg, 2010). Hla has also been demonstrated to activate ADAM10 to cleave vascular endothelial-cadherin present in cell-cell adhesive contacts, which leads to the disruption of the endothelial tissue barrier (Berube and Bubeck Wardenburg, 2013).

Several antibodies targeting Hla are currently being evaluated in clinical trials. KBSA301 is a full human IgG1 antibody that specifically neutralizes Hla and protects host cells from destruction (François et al., 2018). KBSA301 showed an improved microbiologic eradication in patients with hospital-acquired bacterial pneumonia and ventilator-associated bacterial pneumonia, and is currently being investigated in a phase III clinical trial^1^. MEDI4893 is another human monoclonal IgG1 antibody specific for Hla. However, it has recently been reported that MEDI4893 failed to improve the outcome in clinical studies of prevention of *S. aureus* pneumonia in patients in the ICU receiving mechanical ventilation (François et al., 2021). Rouha et al. (2015) generated a human monoclonal antibody (mAb) that cross-reacted with four of the five leukocidins and Hla, which improved protection in murine models of pneumonia and sepsis.

In this study, we aimed to screen for novel specific human mAbs against Hla and evaluated the neutralization function of mAbs *in vitro* and *in vivo*.

## Materials and Methods

### Bacterial Strains

The *S. aureus* strains USA300, 8325-4 and Newman were generously provided by professor Lefu Lan (Department of Molecular Pharmacology, Shanghai Institute of Materia Medica, Chinese Academy of Sciences). The *Escherichia coli* strains DH5α and BL21 were obtained from Novagen. All *S. aureus* cells were streaked onto brain heart infusion (BHI) plates and grown at 37°C for 12 h with shaking at 220 rpm. The *E. coli* cells were grown at 37°C overnight in Luria-Bertani (LB) medium.

### Purification of the Recombinant Proteins

The gene of Hla was amplified from the genome of *S. aureus* 8325-4, and the genes of Hla variants were obtained by overlap PCR with degenerate primers. The PCR products were digested with *Nhe1* and *Xho1*, and then cloned into expression vector pET-28(a). The recombinant plasmid was transformed into *E. coli* strain BL21 (DE3) for expression. The recombinant proteins were induced by isopropyl-beta-D-thiogalactopyranoside (IPTG) and purified with Ni-NTA agarose (GE Healthcare, 17-5318-01). The purified proteins were dialyzed in PBS for 24 h at 4°C and determined by SDS-PAGE (Crowe et al., 1994).

### Hemolytic Activity Assays

The hemolysis activities of wild-type and mutant Hla proteins were measured as previously described with some modifications (Cooper et al., 1966; Yan et al., 2017). In brief, serial dilutions of purified toxins were incubated with 2% (v/v) suspension of washed rabbit erythrocytes at 37°C for 1 h. After incubation, the released hemoglobin in the supernatant was collected by centrifugation, and OD_405_ was measured using a plate reader.

### Selection of scFvs to Hla

Hla-specific scFvs were isolated from a phage display antibody library using the standard procedure. The Hla-immobilized immunotube (Thermo Scientific Nunc^®^, 444202) was blocked with 4% skim milk powder/PBST, then about 10^12^ pfu phages were added into the tube. After incubation, the tube was washed with PBST to remove unbound phages. The conjugated phage was eluted by the addition of 0.1 M pH2.2 Gly-HCl. Totally, three rounds of bio-panning were performed to select phage clones binding to Hla. The single colonies were randomly selected and further screened by monoclonal phage ELISA. The Hla-specific phage clones were analyzed by DNA sequencing.

### Binding Affinity of YG1 Antibody

Binding affinity of YG1 antibody to Hla was determined by ELISA. Briefly, 96-well plates were coated with Hla or its variants individually overnight at 4°C. After blocking, serially diluted antibodies (YG1) were added to wells and incubated at 37°C for 1 h. The horseradish peroxidase/Goat anti-Human antibody (1:40,000; Jackson, 109-035-003) was used to detect the bound antibody.

The YG1 binding kinetics were analyzed using a Biacore X100 instrument. Anti-human IgG antibody was immobilized on the surface of BIAcore’s CM5 sensor chip by a standard coupling protocol. The YG1 antibody was injected over the chip surface at a flow rate of 30 μL/min. Hla (0.85–33 nM in 2.5-fold serial dilutions) were sequentially injected and incubated with YG1 bound on the tips. The association phase was monitored for 180 s and the dissociation phase was monitored for 1,300 s. The Biacore X100 Evaluation software was used to analyze data.

### Binding Specificity of YG1 Antibody

The binding specificity of YG1 to Hla was evaluated by ELISA. Hla, Hlb, and leucocidin subunits (HlgA, HlgB, HlgC, LukD, LukE, and LukF) were coated onto ELISA plates, respectively. YG1 antibody (1 μg/ml) was added to each well and incubated at 37°C for 1 h. The horseradish peroxidase/Goat anti-Human antibody (1:40,000; Jackson, 109-035-003) was used to detect the bound YG1.

### Inhibition of Rabbit RBC Hemolysis

The YG1 antibody was serially diluted and mixed with recombinant Hla or Hla variants (0.06 μg/ml). The mixture was incubated at room temperature for 30 min, then added to 2% (v/v) solution of rabbit erythrocytes. After incubation at 37°C for 1 h, the released hemoglobin in the supernatant was collected, and OD_405_ was determined using a plate reader. The following formula was used to calculate percent inhibition of toxin activity:%inhibition = [(normal activity-inhibited activity)/(normal activity)] × 100%. Data were analyzed with non-linear regression analysis using GraphPad Prism 8.

### A549 Cell-Binding Assay

Flow cytometry was used to assess the binding ability of Hla to A549 cell. 100 μl of A549 cells (5 × 10^5^) were incubated with the 10 μg/ml of Hla or control protein Hlb at room temperature for 1 h. After two washes with PBS, cells were incubated with 100 μl of 5 μg/ml rabbit anti-Hla antibody for 45 min at room temperature. After two washes with PBS, cells were incubated with FITC-conjugated donkey anti-Rabbit antibody (Biolegend, 406403) at room temperature for 30 min. Following two PBS washes, Hla and Hlb bindings were then assessed with an LSRII flow cytometer (BD FACSCalibur, San Jose, United States), and FlowJo Software (Tree Star, Inc., Ashland, OR, United States) was used to analyzed data.

### Inhibition of Hla Binding to A549 Cells

YG1 was serially diluted in 1%BSA/PBS and mixed with Hla at fixed concentrations, and the mixtures were pre-incubated at room temperature for 30 min. YG2 (anti-eLtaS monoclonal antibody), a non-specific human IgG1, was used as a control. The mixtures were then added to A549 cells for 1 h at room temperature. After two washes with PBS, the cells were incubated with 100 μl of 5 μg/ml rabbit anti-Hla antibody for 45 min at room temperature. After two washes with PBS, FITC-conjugated donkey anti-Rabbit antibody was added to A549 cells and incubated for at room temperature 30 min. Following two PBS washes, Hla binding was then assessed with an LSRII flow cytometer (BD FACSCalibur, San Jose, United States), and FlowJo Software (Tree Star, Inc., Ashland, OR, United States) was used to analyze data.

### Acute Peritoneal Infection Murine Model

Six- to eight-week-old female BALB/c mice were intraperitoneally challenged with *S. aureus* 8325-4 (3.8 × 10^8^ CFU/mouse) or USA300 (7.5 × 10^8^ CFU/mouse). Each type of mice was randomized into treatment groups and control groups, each with 16 mice. In the *S. aureus* 8325-4 infection group, 100 μg of YG1 (5 mg/kg) or the IgG1 isotype control YG2 (5 mg/kg) diluted in 200 μl PBS was injected into the peritoneal cavity 4 h prior to the bacterial challenge. In the *S. aureus* USA300 infection group, YG1 or the IgG1 isotype control YG2 at the indicated concentrations was injected intraperitoneally (i.p.) into mice 4 h prior to the bacterial challenge. Mouse survival was monitored over 3 days. To quantify *S. aureus* CFU in kidneys, mice were killed at 48 h postinfection. The kidneys were harvested and homogenized in 1 ml 0.1% Triton X-100. Organ homogenates were serially diluted in PBS and plated on BHI agar plate for triplicate determination of CFU.

### Sublethal Murine Pneumonia Infection Model

Six- to eight-week-old female BALB/c mice were separated into three groups, each consisting of six mice. The mice were passively immunized by i.p. injection of YG1 at the indicated concentrations and then challenged with *S. aureus* 8325-4 (8 × 10^7^ CFU/mouse) *via* the tracheal route. After 72 h postinfection, the infected mice were euthanized and the lung tissues were removed, fixed and stained by H&E.

### Lethal Murine Pneumonia Infection Model

Six- to eight-week-old female BALB/c mice were separated into four groups, each consisting of 16 mice. The mice were passively immunized by i.p. injection of YG1 at the indicated concentrations or the IgG1 isotype control YG2 and then challenged 24 h later with *S. aureus* USA300 (2.3 × 10^9^ CFU/mouse) into the left nares. Animals were placed supine into a cage for recovery, and survival was monitored over 3 days.

### Bacteremia Infection Murine Model

Six- to eight-week-old female BALB/c mice were separated into three groups, each consisting of 16 mice. The mice were passively immunized by i.p. injection of YG1 or the IgG1 isotype control YG2 (5 mg/kg) and then challenged 4 h later by retroorbital injection of the lethal dose of *S. aureus* Newman (2.5 × 10^7^ CFU/mouse). Mouse survival was monitored over 2 weeks. To quantify *S. aureus* CFU in kidneys, mice were killed at 14 days postinfection. The kidneys were harvested and homogenized in 1 ml 0.1% Triton X-100. Organ homogenates were serially diluted in PBS and plated on BHI agar plate for triplicate determination of CFU.

### Homology Modeling

Firstly, the 3-D theoretical structure of Hla was modeled using Homology program (Joshi et al., 2010). The fragment variable (Fv) region of the anti-Hla, named as YG1, was constructed using Modeler program (InsightII, 2000). Dealing with the protein 3-D structures Database (PDB), the suitable protein structures were chosen as template. Based on the primary sequence alignment and the computer-guided molecular modeling, the 3-D theoretical conformations of the proteins were optimized with the steepest descent and conjugate gradient methods under CVFF forcefield. All calculations were performed on IBM workstation with a distance dependent dielectric constant and a long range non-bonded cutoff of 8 Å.

### Rigid Body Docking of Hla and YG1

Based on connelly’s MS program, the possible interaction sites on Hla were assigned with the 3-D spatial surface and generated spheres to fill the cavities. According to the docking rules and overlapping spheres cluster criteria, the individual spheres were then clustered. Under CVFF forcefield, the potential complex structure of Hla and YG1 was constructed using Dock program. Using van der waals interaction and columbic energy, the docked orientations were evaluated. And then, dealing with continuum dielectric method, each docked orientation was subjected to quasi-Newton optimization.

### Statistical Analyses

Data were presented as the mean ± SD from at least three independent experiments. A two-tailed Student’s *t* test was used to assess significant differences between groups. Survival was described using the Kaplan–Meier curves and compared by the log-rank test. All statistical analyses were performed with GraphPad Prism software (version 8.00). Values of *p* less than 0.05 was considered as statistically significant.

### Ethics Statement

All animal experiments were conducted in accordance with protocols approved by the Animal Care and Use Committee of Beijing Institute of Basic Medical Sciences, with the approval number BMS-111248.

## Results

### Selection of Anti-Hla Human mAbs

**FIGURE 1:**
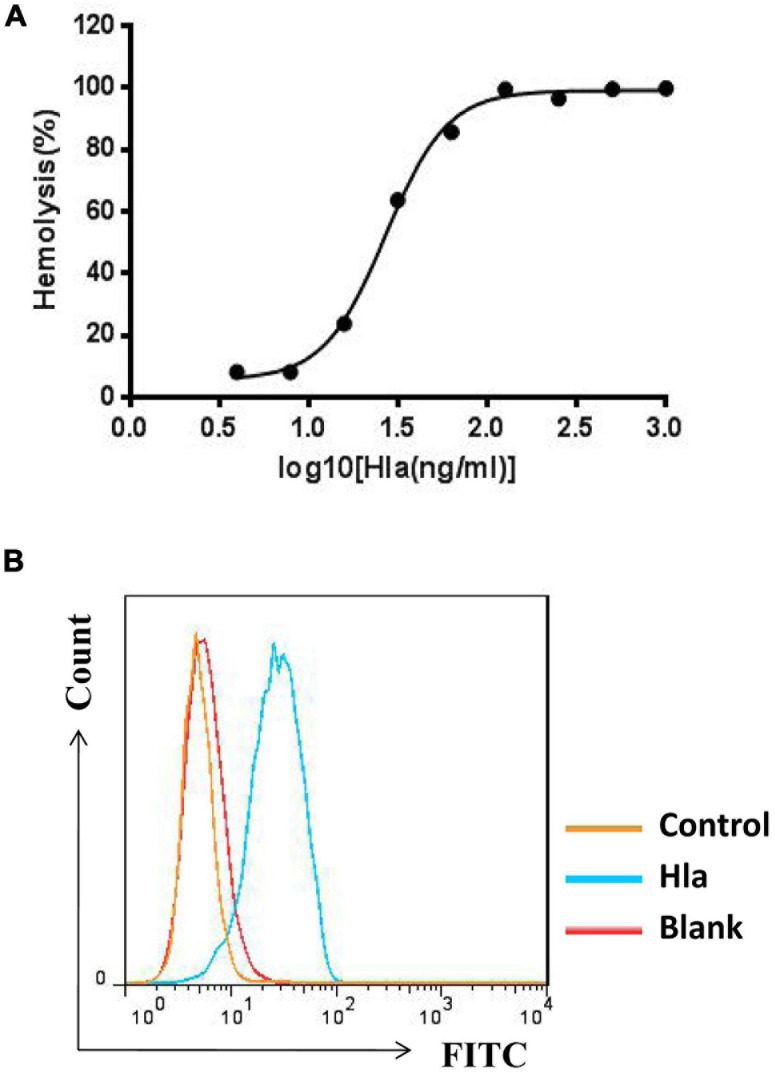
Measurement of Hla activities. **(A)** Effects of HIa on the lysis of rabbit red blood cells. Serial dilutions of Hla (0, 0.0156, 0.0313, 0.0625, 0.125, 0.25, 0.5, 1, and 2 μg/mL) were each incubated with rabbit red blood cells for 1 h at 37°C. The samples were centrifuged, and the absorbance of the supernatants was measured at 405 nm. **(B)** Hla bound to A549 cells. A549 cells (5 × 10^5^) were incubated with HIa or control protein (10 μg/mL) for 1 h. The binding of proteins was detected by FCM with rabbit anti-Hla antibodies and FITC-conjugated donkey anti-rabbit antibodies.

To screen for specific mAbs against Hla, the recombinant protein was expressed and purified (Supplementary Figure 1), and the activities of Hla were subsequently determined. We found that Hla could lyse rabbit red blood cells (RBCs) and bind to A549 cells (Figures 1A,B). We generated a full human naïve Fab phage library with an estimated diversity of 1.81 × 10^9^, which was subsequently screened for antibodies against Hla. After three rounds of bio-panning, 42 phage clones targeting Hla were isolated, and 18 clones were further characterized by nucleotide sequencing (Supplementary Figure 2). Only one antibody that specifically bound to Hla (designated YG1) was selected from the library. To identify the germline variable region genes for YG1, sequence analysis was performed using the IMGT database^2^. The heavy chain V region sequence (V_H_) of YG1 contained mutations in 32 residues compared with that of the IGHV1-24 × 01 family member, and the light chain V sequence (V_L_) contained mutations in 20 residues compared with the that of IGKV3-15 × 01 germline kappa sequence.

### Evaluation of YG1 Function *ex vivo*

**FIGURE 2:**
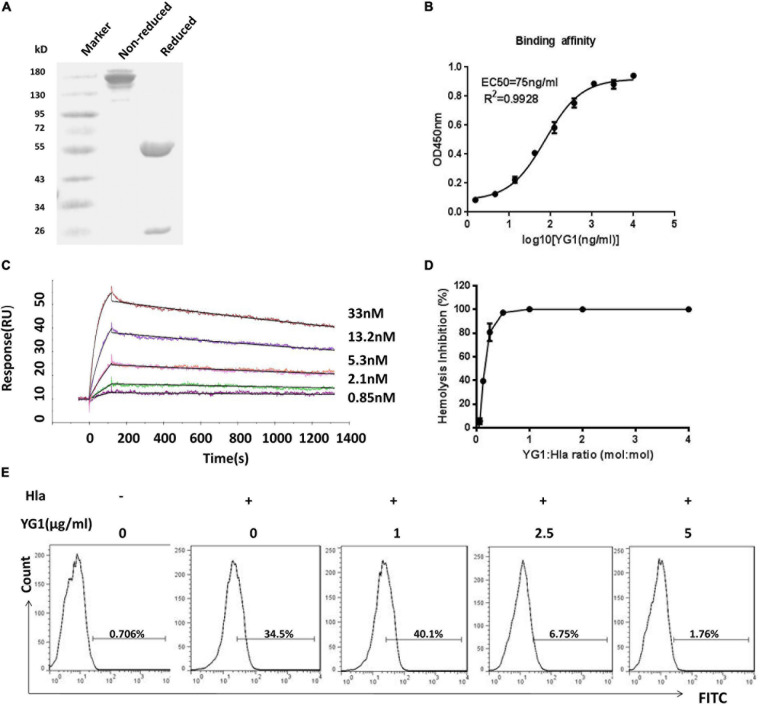
Evaluation of YG1 function *ex vivo*. **(A)** SDS-PAGE analysis of the purified YG 1 mAb under reducing and nonreducing conditions. **(B)** The binding affinity of YG1 to Hla was determined by ELISA. Hla protein was coated onto 96-well plates, and YG1 was added at various concentrations. Bound IgG was detected using horseradish peroxidase (HRP)-conjugated goat anti-human IgG. **(C)** Antibody affinity of YG1 to Hla was determined with surface plasmon resonance (BlAcore). Anti-human IgG antibody was immobilized on the carboxymethylated dextran surface of a CM5 chip, and YG 1 was captured by the immobilized antibody. Hla protein was injected at the indicated concentrations. The data were analyzed using BlAcore X100 Evaluation software. **(D)** YG1 inhibited the hemolysis of red blood cells (RBCs) in a concentration-dependent manner. Serial dilutions of YG1 were incubated with Hla for 30 min at 37°C. The mixture was further incubated with rabbit RBCs for 1 h at 37°C. The samples were centrifuged, and the absorbance of the supernatants was measured at 405 nm. **(E)** YG1 inhibited Hla binding to A549 cells. Serial dilutions of YG1 (0, 1, 2.5, and 5 μg/mL) were incubated with Hla for 30 min at 37°C. The mixture was further incubated with A549 cells (5 × 10^5^) for 1 h. The binding of Hla was detected by FCM with rabbit anti-Hla antibodies and FITC-conjugated donkey anti-rabbit antibodies.

To test the functions of YG1, the mAb was expressed and purified as a full-length human IgG1 antibody (Figure 2A). The binding affinity of YG1 to Hla was subsequently evaluated by ELISA and surface plasmon resonance. We found that YG1 had an EC50 of 75 ng/mL and a KD value of 2.6 ± 0.348 nM (Figures 2B,C and Table 1). To evaluate the specificity of YG1 for Hla, YG1 binding to Hla, Hlb, and leucocidin subunits (HlgA, HlgB, HlgC, LukD, LukE, and LukF) was evaluated. ELISA results showed that YG1 bound only to Hla but not to the other extracellular toxins (Supplementary Figure 3). Thus, we further investigated whether YG1 could bind to native Hla. Through western blot analysis, we demonstrated that YG1 could bind to Hla in 8325-4 supernatants (Supplementary Figure 4). Accordingly, we then assessed the inhibitory activity of YG1 through Hla hemolytic assays. YG1 was titrated from 600 to 4.7 ng/mL in the presence of constant amounts of Hla and RBCs. The results showed that YG1 inhibited Hla-mediated RBC lysis and exhibited 50% inhibition at a 0.15:1 (IgG:Hla) molar ratio, indicating that YG1 inhibited pore formation in rabbit RBCs (Figure 2D). Based on these results, we further investigated the effects of YG1 on Hla binding to A549 cells. Hla was treated with different concentrations of YG1 or the isotype control prior to incubation with A549 cells. Flow cytometry was then used to detect Hla binding. We found that YG1 was able to block Hla binding to A549 cells in a concentration-dependent manner (Figure 2E and Supplementary Figure 5).

**TABLE 1:** Association and dissociation rate constants and apparent binding constants for YG1 MAb.

**Sample**	**Kon (1/Ms)**	**Koff (1/s)**	**K_D_(M)**	**Average K_D_(nM)**
YG1	1.328E + 5	2.552E-4	1.922E-9	
	9.115E + 4	2.715E-4	2.979E-9	2.6 ± 0.348
	9.264E + 4	2.736E-4	2.953E-9	

### Assessment of the Protective Effects of YG1 *in vivo*

**FIGURE 3:**
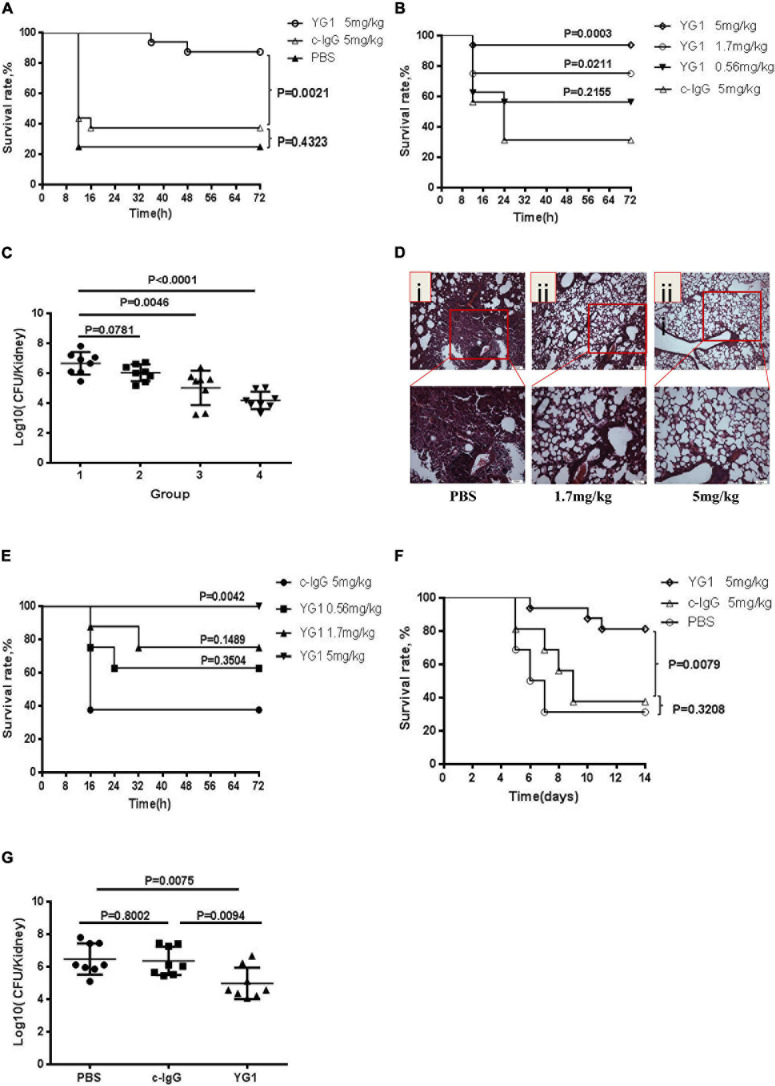
Assessment of the protective effects of YG1 *in vivo*. **(A,B)** Assessment of the antimicrobial effects of YG 1 in a murine model of acute peritoneal infection. YG 1 or c-IgG was injected into the peritoneal cavity of BALB/c mice (*n* = 16) 4 h prior to challenge with *S. aureus* 8325-4 (3.8 × 10^8^ CFU/mouse) **(A)**. YG I or c-IgG at the indicated concentrations was injected into the peritoneal cavity of BALB/c mice (*n* = 16) 4 h prior to challenge with USA300 (7.5 × 10^8^ CFU/mouse) **(B)**. The survival rates were measured at different time points postchallenge. Data are presented as the percentage of mice surviving. Survival curves were determined using the Kaplan–Meier method and compared using the log-rank test. **(C)** Bacterial colonies in kidneys were counted at 48 h postinfection [(1) c-IgG 5 mg/kg, (2) YG1 0.56 mg/kg, (3) YG I 1.7 mg/kg, and (4) YG1 5 mg/kg]. Data are representative of three independent experiments and shown as the mean ± SD. **(D,E)** YG1 protected mice from *S. aureus* infection in a pneumonia infection model. YG1 was injected into the peritoneal cavity of BALB/c mice (*n* = 6) 4 h prior to challenge with S. aureus 8325-4 cells (8 × 107 CFU/mouse). Lung sections were collected at 72 h postchallenge and stained with hematoxylin-eosin **(D)**. YG1 or c-IgG at the indicated concentrations was injected into the peritoneal cavity of C57BL/6J mice (n = 8) 24 h prior to challenge with *S. aureus* USA300 (2.3 × 109 CFU/mouse). The survival rates were measured at different time points postchallenge **(E)**. **(F)** Determination of the effects of YG I in a bacteremia model. YG I or c-IgG was injected into the peritoneal cavity of BALB/c mice (*n* = 16) 4 h prior to intravenous challenge with *S. aureus* Newman (2.5 × 10^8^ CFU/mouse). The survival rates were measured at different time points postchallenge. Data are presented as the percentage of mice surviving. Survival curves were determined using the Kaplan–Meier method and compared using the log-rank test. **(G)** Bacterial colonies in kidneys were counted at 14 days postinfection. Data are representative of three independent experiments and shown as the mean ± SD. Significant differences between groups were evaluated using two-tailed student’s *t* tests.

Next, the protective effects of YG1 were assessed in a murine model of acute peritoneal infection. In this experiment, YG1 or the IgG1 isotype control YG2, was administered i.p. at 100 μg/mouse (5 mg/kg) 4 h prior to a lethal challenge with *S. aureus* 8325-4. YG1 administration resulted in significantly increased survival of mice over the 3-day experiment (Figure 3A), indicating that YG1 protected mice from *S. aureus* infection. To determine whether YG1 has a protective effect against severe disease caused by diverse clinical isolates, Balb/c mice were passively immunized i.p. with YG1 (at 5, 1.7, or 0.56 mg/kg) or YG2 (5 mg/kg) 4 h before i.p. infection with USA300 (7.5 × 10^8^ CFU/mouse). YG1 administration resulted in increase in survival of mice in a dose-dependent manner (Figure 3B).

To better understand the effects of YG1 prophylaxis on the pathogenesis of *S. aureus* peritoneal infection, the bacterial load in the kidneys was determined by plating serial dilutions on BHI 48 h post-infection. Infected mice passively immunized with YG1 exhibited a dose-dependent reduction in bacterial CFU in the kidneys (Figure 3C). The YG1 protective effect was further evaluated in a staphylococcal murine-pneumonia model. In this sublethal murine pneumonia model, YG1 (at 5 or 1.76 mg/kg) was injected i.p. 4 h prior to *S. aureus* 8325-4 infection. At 72 h post-challenge, we performed a histopathological analysis of the lungs of the mice and found that *S. aureus*-induced tissue damage was decreased by YG1 prophylaxis (Figure 3D). In the lethal murine pneumonia model, Balb/c mice were passively immunized i.p. with YG1 (at 5, 1.7, or 0.56 mg/kg) or YG2 (5 mg/kg) and infected 24 h later with a lethal dose of USA300 (2.3 × 10^9^ CFU/mouse). YG1 administration (5 mg/kg) significantly increased the survival of mice relative to the IgG1 isotype control YG2 (Figure 3E). We also evaluated the effects of YG1 in a murine model of *S. aureus* bacteremia. Balb/c mice were passively immunized i.p. with YG1 or the IgG1 isotype control YG2 (5 mg/kg). Mice were challenged 4 h later by intravenous injection of a lethal dose of *S. aureus* Newman (2.5 × 10^8^ CFU/mouse). Survival was monitored over 14 days (Figure 3F), and the number of bacterial colonies in the kidneys of surviving mice was determined. Notably, passive immunization of YG1 significantly decreased the colonization of *S. aureus* in the kidney (Figure 3G). These results further confirmed that anti-Hla YG1 mAb is functional *in vivo*.

### Mapping the Epitope of Hla Recognized by YG1 Using Hla Variants

Using the alignments (Supplementary Figures 6A,B), the model of the 3D structure of V_H_ and V_L_ was obtained from the PDB database. The search for the templates in the PDB database was operated according to the Blosum62 matrix of the BLAST program. The most suitable template structures for the framework of V_H_ and V_L_ were assigned. The V_H_ fragment of YG1 has ∼68% sequence identity with 5aze (PDB code, Fab fragment antibody binding to calcium-dependent antigen, 6RL#9), and the lengths of CDR1 and CDR2 between YG1 and 5aze are the same (Hironiwa et al., 2016). The V_L_ fragment of YG1 has ∼82% sequence identity with neutralizing antibody 17b target to GP120 (PDB code, 1g9m), and the lengths of the corresponding CDR1 and CDR2 are identical (Kwong et al., 2000). The CDR3s in the V_H_ and V_L_ of YG1 had different lengths from the templates. Using a computer-guided molecular modeling method, considering the orientation of the model, the theoretical 3D conformations of V_H_ and V_L_ of YG1 were constructed and minimized (Supplementary Figures 7A,B). With the Procheck program, analysis of the two structures showed that 98% of the non-glycine and non-proline residues adopted either the most favorable or additional allowed regions on a Ramachandran plot. The results showed that the theoretical 3D structures of the V_H_ and V_L_ were credible.

**FIGURE 4:**
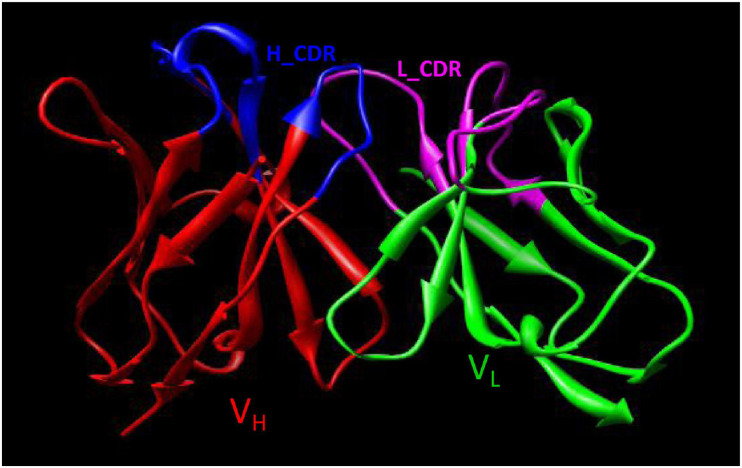
The 3D Fv structure of YG1 derived from computer-guided homology modeling and molecular docking methods. The red ribbon denotes the main carbon atom orientation of VH, the green ribbon denotes that of VL, the blue ribbon denotes the CDR of VH, and the pink ribbon denotes the CDR of VL.

**FIGURE 5:**
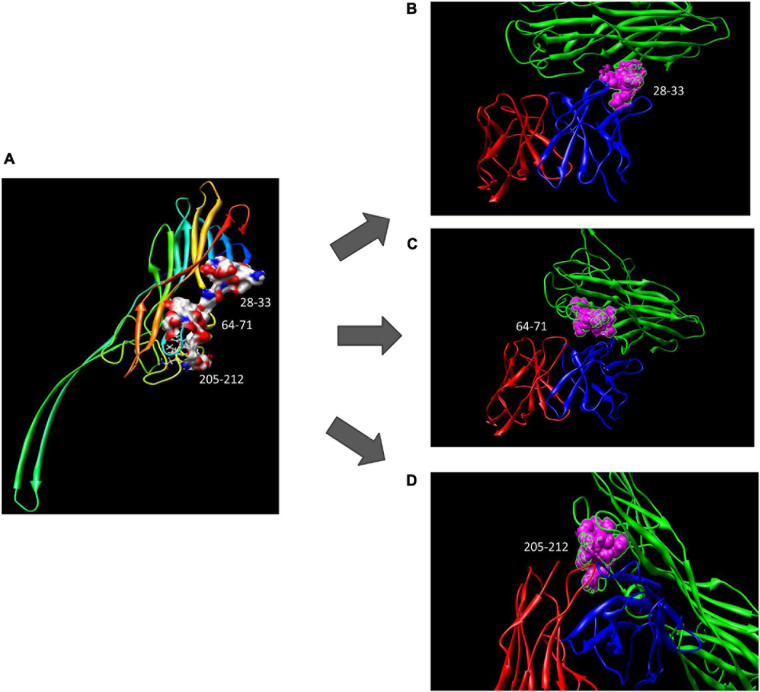
The potential epitope distribution in Hla and the theoretical interaction mode between Hla and YG1. **(A)** The potential epitope distribution in Hla, i.e., the hydrophobic and hydrophilic map of the epitopes (including 28–33, 64–71, and 205–212). **(B)** The interaction mode between the epitope at positions 28–31 and YG 1. Green denotes Hla, red denotes VL, and blue denotes VH. **(C)** The interaction mode between the epitope at 64–71 and YG1. Green denotes Ma, red denotes VL, and blue denotes VH. **(D)** The interaction mode between the epitope at 205–212 and YG1. Green denotes Hla, red denotes VL, and blue denotes VH.

Using computer-guided molecular docking, the 3D structure of the Fv fragment of YG1 was modeled (Figure 4). Moreover, the CDRs of the V_H_ and V_L_ of YG1 formed a pocket, which possessed a strong hydrophobic surface, allowing binding with the antigen epitope. According to the X-ray crystal structure of Hla, the solvent-accessible surface distribution of Hla was analyzed. The three potential epitopes (i.e., 28–33, 64–71, and 205–212) of Hla, which possessed strong hydrophobic surfaces, were predicted. Furthermore, the 3D complex structure of the Hla and Fv fragment of YG1 was constructed using molecular docking (Supplementary Figure 8). The interaction model between the potential epitopes of Hla and the Fv fragment of YG1 is shown in Figure 5. Notably, the potential epitope from positions 28 to 33 of Hla bound YG1 with weak binding energy (−8.31 kcal/mol), and the epitope from positions 64 to 71 of Hla bound YG1 with an energy of −7.42 kcal/mol. However, the epitope from positions 205 to 212 of Hla bound YG1 with −42.86 kcal/mol. These results showed that the epitope from positions 205 to 212 of Hla was the major domain recognized by YG1.

**FIGURE 6:**
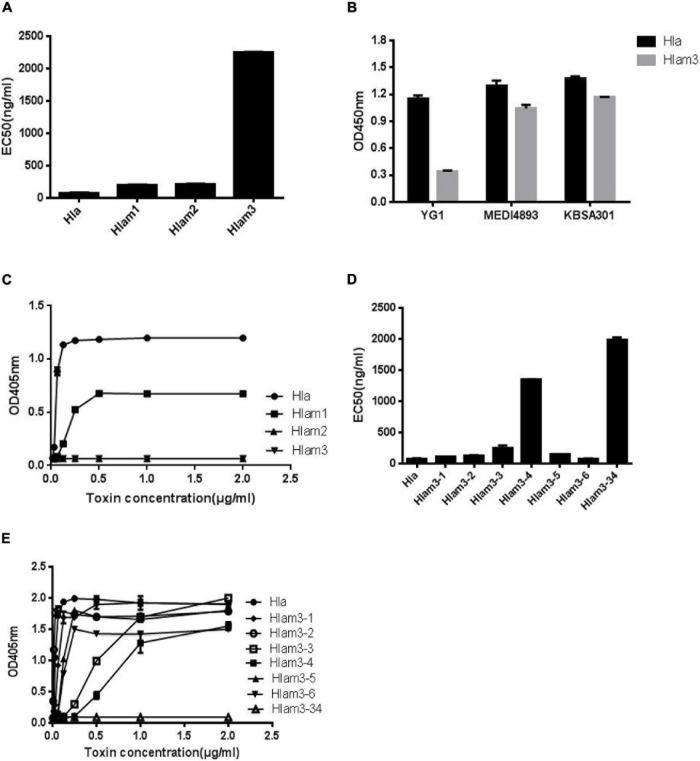
Mapping the epitope of Hla recognized by YG1 using Hla variants. **(A,D)** The binding affinity of YG1 to Hla and its mutations was determined by ELISA. Hla or Hla mutants were coated onto 96-well plates, and YG I was added at various concentrations. Bound YG I was detected using horseradish peroxidase (HRP)-conjugated goat anti-human IgG. The EC50 value was calculated using GraphPad Prism Software. **(B)** The binding affinities of YG1, MEDI4893, and KBSA301 to Hla or Hlam3 were determined by ELISA. Hla or Hlam3 protein was coated onto 96-well plates, and antibodies were added at various concentrations. Bound IgG was detected using horseradish peroxidase (HRP)-conjugated goat anti-human IgG. **(C,E)** Effects of Hla and Hla mutants on the lysis of rabbit red blood cells. Serial dilutions of Hla or Hla mutants (0, 0.015625, 0.03125, 0.0625, 0.125, 0.25, 0.5, 1, and 21 μg/mL) were incubated with rabbit red blood cells for 1 h at 37°C. The samples were centrifuged, and the absorbance of the supernatants was measured at 405 nm.

To further identify the epitope of YG1 on Hla, three Hla variants, Hlam1, Hlam2, and Hlam3, were constructed by replacing amino acids 28–33, 64–71, and 205–212 of Hla with alanine, respectively (Supplementary Figure 9A). The Hla mutants were subsequently expressed and purified (Supplementary Figure 9B), and the binding of YG1 to these variants was characterized through ELISA. We found that YG1 bound to Hlam1, Hlam2, and Hlam3 mutants with different affinity EC50 values of 197, 215, and 2,255 ng/mL, respectively, and the mutation in 205–212 (Hlam3) dramatically decreased the binding of YG1 (Figure 6A and Supplementary Figure 9C). We next measured the binding affinities of MEDI4893 and KBSA301 to Hla or Hlam3 by ELISA and found that Hlam3 induced a slight reduction in MEDI4893 and KBSA301 binding compared with Hla (Figure 6B). We further assessed the hemolytic activity of Hla mutations using a rabbit RBC lysis assay. Notably, Hlam2 and Hlam3 mutations were devoid of hemolytic activity, whereas Hlam1 exhibited 27% of the specific activity of wild-type Hla (Figure 6C).

Given that mutations in 205–212 resulted in decreased YG1 binding and hemolytic activities, we further characterized the essential amino acids in this region. Six Hla variants (Hlam3-1, Hlam3-2, Hlam3-3, Hlam3-4, Hlam3-5, and Hlam3-6) were designed by replacing amino acids K205, D208, N209, F210, L211, and D212 of Hla with alanine, respectively (Supplementary Figure 10A). The Hla mutants were subsequently expressed and purified (Supplementary Figure 10B), and the binding of YG1 to these variants and the hemolytic activities of these variants were then assessed. We found that substitution of N209 and F210 of Hla impaired YG1 binding compared with other amino acids (Figure 6D and Supplementary Figure 10C). Moreover, Hlam3-3 and Hlam3-4 mutations caused obvious reductions in hemolytic activities (Figure 6E).

We further constructed an Hla variant (Hlam3-34) by replacing amino acids N209 and F210 of Hla with alanine (Supplementary Figure 10A). The Hla mutant was subsequently expressed and purified (Supplementary Figure 10B), and the YG1 binding ability and hemolytic activities of Hlam3-34 were determined. The double amino acid mutation induced greater reductions in YG1 binding than Hlam3-3 and Hlam3-4 (Figure 6D and Supplementary Figure 10C). Moreover, we found that Hlam3-34 showed an almost complete loss of hemolytic activity (Figure 6E).

## Discussion

Infection caused by multidrug-resistant *S. aureus* has become a serious threat to public health worldwide. The development of new antibiotics against *S. aureus* have not kept pace with the evolution of antimicrobial resistance (Tavares et al., 2013; Antonanzas et al., 2015; Monaco et al., 2017), which has led researchers to explore pathogen-specific methods, including mAbs targeting *S. aureus* and its virulence determinants to prevent or treat *S. aureus* infections (Hua et al., 2015; Tkaczyk et al., 2017).

As a pore-forming toxin, Hla is produced by most pathogenic strains of *S. aureus* and plays an essential role in the pathogenesis of pneumonia, sepsis, and endocarditis (Kebaier et al., 2012; Berube and Bubeck Wardenburg, 2013; Powers et al., 2015). Because of the major role of Hla in these diseases, it is currently being developed as an mAb target. In this study, we developed a human mAb (YG1) against Hla, which was capable of neutralizing Hla. We found that YG1 could prevent Hla-mediated RBC lysis, exhibiting 50% inhibition at a 0.15:1 mAb:toxin molar ratio. *In vivo* studies showed that administration of YG1 protected mice against two methicillin-sensitive *S. aureus* strains (8325-4 and Newman) and one methicillin-resistant *S. aureus* strain (USA300).

We also constructed and analyzed the 3D complex structure of Hla and the Fv fragment of YG1. The structure of the Hla-YG1 Fv complex revealed that the epitope from positions 205 to 212 of Hla was important for YG1 binding as this epitope could bind both the V_H_ and V_L_ of YG1, whereas the epitopes from positions 28 to 33 and positions 64 to 71 of Hla could only bind the V_L_ of YG1. We constructed various Hla variants to further characterize the YG1 epitope of Hla and found that replacing the residues 205–212 of Hla with alanine negatively impacted YG1 binding. We further demonstrated that amino acids N209 and F210 of Hla were functionally and structurally important for YG1 binding.

Oganesyan et al. (2014) reported that MEDI4893 recognized the two epitopes of Hla: Asn-177 to Arg-200 and Thr-261 to Lys-271. We found that YG1 recognized the amino acids 205–212 of Hla. YG1 recognized a novel epitope on the rim region of Hla. Hla exerts virulence upon binding to the specific cellular receptor (ADAM10) on target cell membranes such as platelets, endothelial cells, and immune cells (Powers and Bubeck Wardenburg, 2014). We found that YG1 could block Hla binding to A549 cells in a concentration-dependent manner, suggesting that YG1 inhibited Hla binding to ADAM10. Arg-200 is an important part of the interaction with ADAM10, as well as those amino acids close to positions 205 to 212. The YG1/Hla complex might prevent Hla interaction with its receptor.

In conclusion, we generated a novel anti-Hla human mAb, YG1, which dramatically reduced *S. aureus* infection. We further characterized the epitope of YG1 directed against Hla. YG1 bound to the target epitope from positions 205 to 212 of Hla, in contrast to MEDI4893 and KBSA301. The neutralizing antibody YG1 may be an alternative approach to the currently available treatments for the prevention and management of serious *S. aureus* infections.

## Data Availability Statement

The original contributions presented in the study are included in the article/Supplementary Material, further inquiries can be directed to the corresponding author/s.

## Author Contributions

GY and JF: conceptualization and project administration. FL: methodology and writing – original draft preparation. ZG and YL: validation. JL and CL: formal analysis. YG and YM: resources. GY: writing – review and editing and funding acquisition. BS: supervision. All authors contributed to the article and approved the submitted version.

## Conflict of Interest

The authors declare that the research was conducted in the absence of any commercial or financial relationships that could be construed as a potential conflict of interest.

## Footnotes

1 https://clinicaltrials.gov/ct2/show/NCT03816956

2 http://www.imgt.org/
