# Supplementary material for: Identification of a Human Anti-Alpha-Toxin Monoclonal Antibody Against Staphylococcus aureus Infection
Source: Front Microbiol. 2021 Jul 15;12:692279. doi: 10.3389/fmicb.2021.692279 (PMC8319846; doi:10.3389/fmicb.2021.692279)
Supplement: Supplementary file 1 [file Data_Sheet_1.PDF]

Supplementary Material

Figure S1

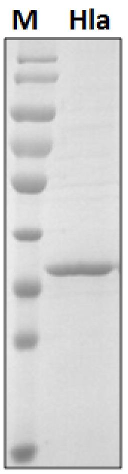

Figure S1. SDS-PAGE analysis of the purified Hla protein under reducing conditions.

Figure S2

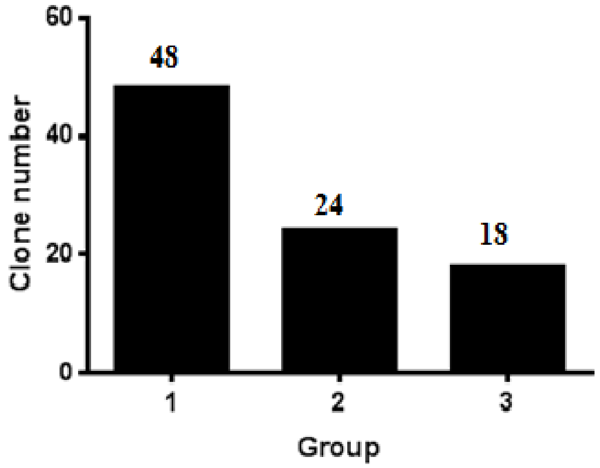

Figure S2. Selection of Hla neutralizing antibodies from a naïve human Fab phage library. After three rounds of panning, 90 clones were screened by ELISA for Hla binding, and 42 clones could bind specifically to Hla (1. $OD_{450} < 1$, 2. $OD_{450} = 1–1.5$, 3. $OD_{450} > 1.5$).

Figure S3

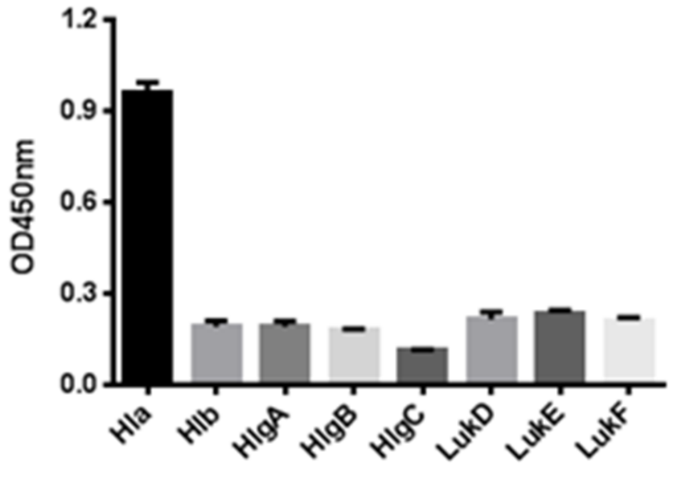

Figure S3. The binding specificity of YG1 for Hla was evaluated by ELISA. Eight different toxins were coated onto 96-well plates, and YG1 was added. Bound YG1 was detected using peroxidase (HRP)-conjugated goat anti-human IgG.

Figure S4

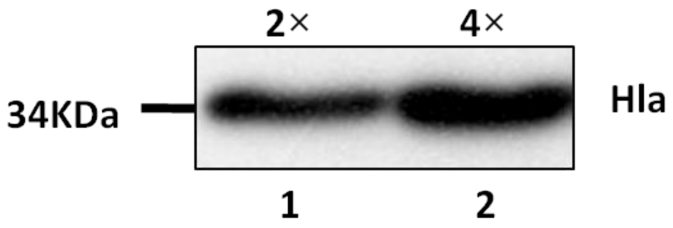

Figure S4. The binding ability of YG1 to native Hla was determined using western blotting. The supernatants from *S. aureus* 8325-4 at 2× and 4× concentrations were subjected to SDS-PAGE on 12% gels, followed by transfer to PVDF membranes. The membranes were then incubated with YG1 (1 μg/mL). The binding of YG1 was detected using horseradish peroxidase (HRP)-conjugated goat anti-human IgG.

Figure S5

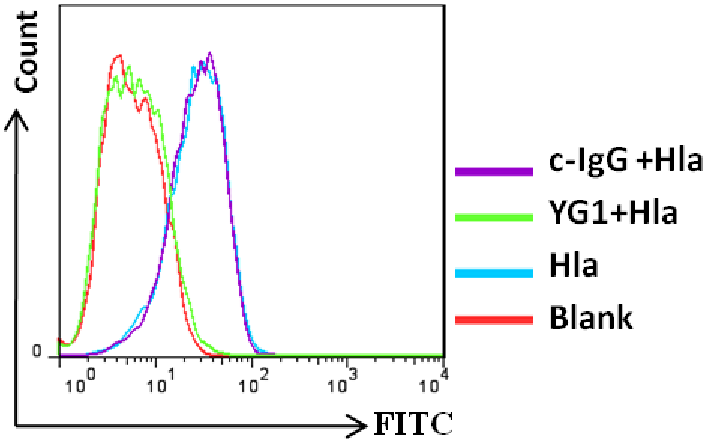

Figure S5. YG1 inhibited Hla binding to A549 cells. Hla was incubated with YG1 for 30 min. The mixture was further incubated with A549 cells (5×10^5) for 1h. The binding of Hla was detected by FCM with rabbit anti-Hla antibodies and FITC-conjugated donkey anti-rabbit antibodies.

Figure S6

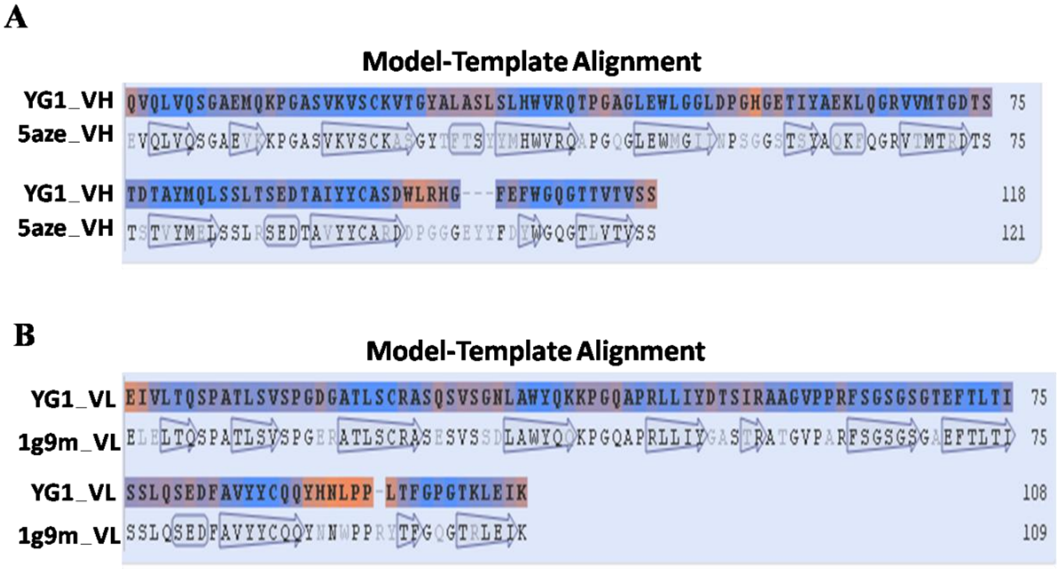

Figure S6. Sequence alignment between YG1 and the 3D model derived from PDB database using the BLASTp program (http://www.ncbi/nlm.nih.gov/blast).(A) Sequence comparison of VH between YG1 and the best match model 5aze (PDB code). (B) Sequence comparison of VL between YG1 and the best match model 1g9m (PDB code).

Figure S7

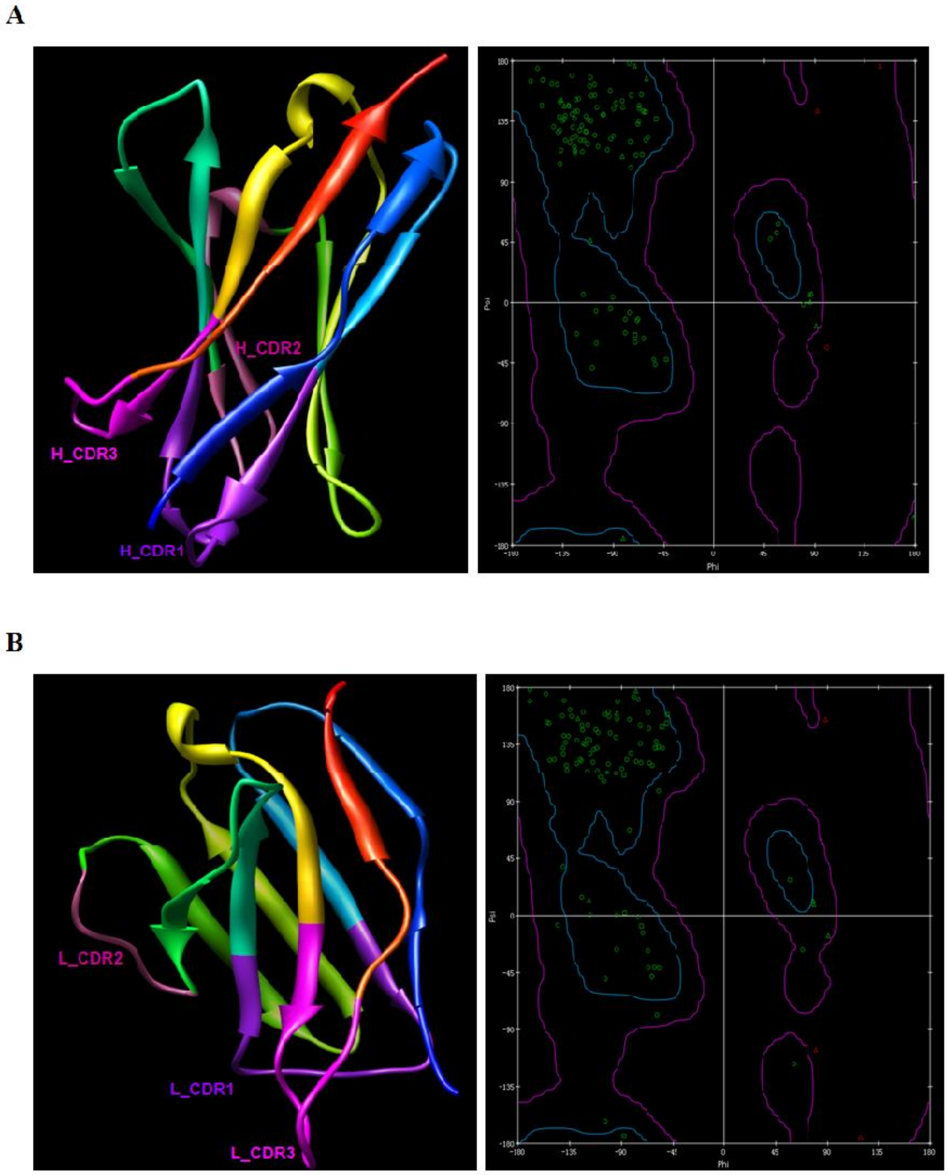

Figure S7. The 3D modeling structures and Ramachandran maps of VH (A) and VL (B) of YG1.

Figure S8

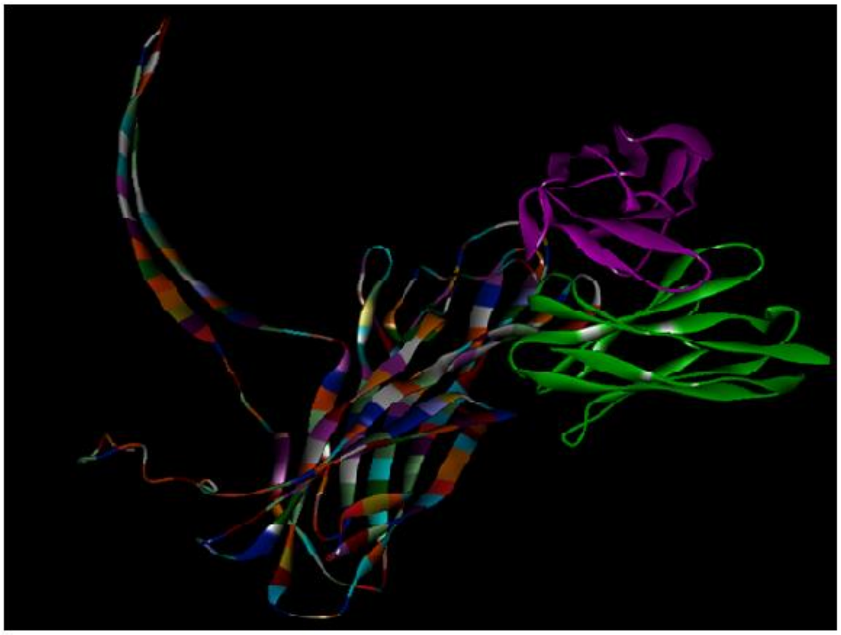

Figure S8. The 3D modeling complex structures of YG1 and Hla. The colored ribbon denotes the main chain carbon atom orientation of Hla. The pink ribbon denotes VH, and the green ribbon denotes VL of YG1.

Figure S9

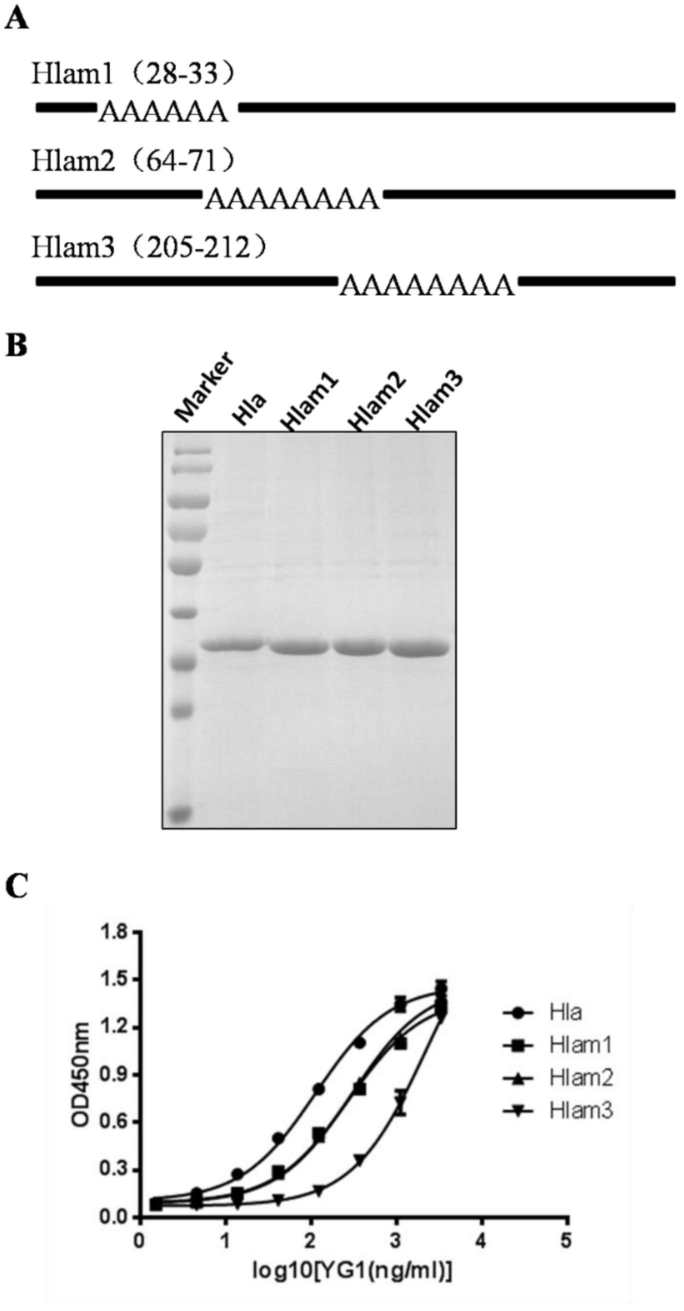

Figure S9. Generation of Hla variants proteins and measurement of the binding affinity of YG1 to Hla and Hla mutants. (A) Schematic diagram of Hla mutants. Amino acids 28–33, 64–71, and 205–212 of Hla were replaced with alanine. (B) SDS-PAGE analysis of the purified Hla mutants under reducing conditions. (C) The binding affinity of YG1 to Hla and Hla mutants was determined by ELISA.

Figure S10

A

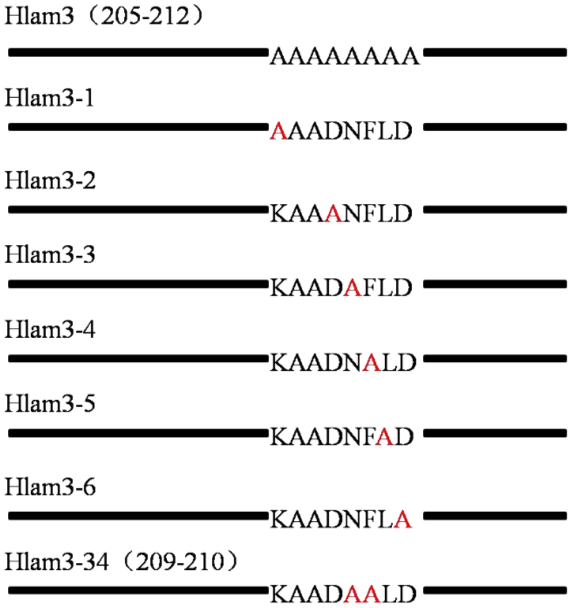

B

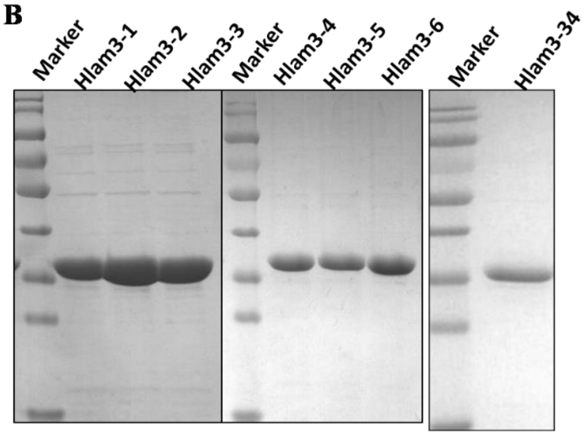

C

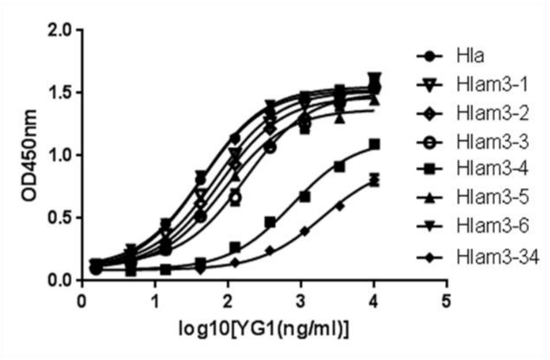

Figure S10. Generation of Hla variants proteins and measurement of the binding affinity of YG1 to Hla and Hla mutants. (A) Schematic diagram of Hla mutants. Amino acids K205, D208, N209, F210, L211, and D212 of Hla were replaced with alanine. (B) SDS-PAGE analysis of the purified Hla mutants under reducing conditions. (C) The binding affinity of YG1 to Hla and Hla mutants was determined by ELISA.
